# Mobile psychiatry: towards improving the care for bipolar disorder

**DOI:** 10.1186/1752-4458-6-5

**Published:** 2012-05-29

**Authors:** Pawel Prociow, Katarzyna Wac, John Crowe

**Affiliations:** 1 Electrical Systems and Optics Research Division, Faculty of Engineering, University of Nottingham, Nottingham, UK; 2 Institute of Services Science, University of Geneva, Geneva, Switzerland; 3 Electrical Systems and Optics Research Division, Faculty of Engineering, University of Nottingham, Nottingham, UK

**Keywords:** Mental health, Personalized monitoring, Bipolar disorder, Pervasive monitoring

## Abstract

**Background:**

Mental health has long been a neglected problem in global healthcare. The social and economic impacts of conditions affecting the mind are still underestimated. However, in recent years it is becoming more apparent that mental disorders are a growing global concern and there is a necessity of developing novel services and researching effective means of providing interventions to sufferers. Such novel services could include technology-based solutions already used in other healthcare applications but are yet to make their way into standard psychiatric practice.

**Methods:**

This manuscript proposes a system where sensors are utilised to devise an “early warning” system for patients with bipolar disorder. The system, containing wearable and environmental sensors, would collect behavioural data independent from the patient’s self-report. To test the feasibility of the concept, a prototype system was devised, which was followed by trials including four healthy volunteers as well as a bipolar patient.

**Results:**

The sensors utilised in the study yielded behavioural data which may be of significant use in detecting early effects of a bipolar episode. Basic processing performed on particular data inputs provided information about activity patterns in areas, which are usually strongly influenced by the course of Bipolar Disorder.

**Conclusions:**

The manuscript discusses the basic usage issues and other barriers which are to be tackled before technology-based approaches to mental care can be successfully rolled out and their true value appraised.

## Introduction

Mental health has long been a neglected problem in global healthcare. The social and economic impacts of conditions affecting the mind are still underestimated, much as the problems of those who suffer from such illnesses were overlooked and their burden trivialised. However, in recent years it is becoming more apparent that mental disorders are a growing concern not only in the “developed world” but globally as well.

When considered in terms of mortality, mental disorders are not to be found among the main priorities of public health [1,2]. This is largely due to the fact that the vast majority of neuropsychiatric conditions (even if untreated) do not directly lead to death of the patient. Nevertheless in extreme circumstances these illnesses can increase the risk of mortality (e.g. by suicide) [2]. However, reports, which utilize more complex measures of quality of life to calculate the burden of diseases, rank chronic mental conditions almost as high as respiratory and cardiovascular conditions and higher than all types of cancer and HIV. Moreover, it is projected that by 2030 mental disorders will constitute 15% of the global disease burden with unipolar depression becoming the second highest occurring condition [2]. Considering the rising burden of such conditions, most health organisations and other policy-makers, in their reports, point to the necessity of developing novel services and researching non-costly and effective means of providing interventions to sufferers [3].

Currently, clinical psychiatry (and psychology) relies greatly on the retrospective self-reporting of patients’ symptoms. Such methods of data collection, whether it is an end-of-day paper diary, simple interview or a structured questionnaire, have one common feature in that the information must be recalled from the patient’s memory [4]. This may lead to misjudgement of the patient’s state as studies show that gathering information retrospectively is subject to multiple systematic distortions and biases (e.g. towards socially acceptable answers) [5]. It is also known that “positive” events are more likely to be remembered than ones with negative association for the respondent. The processing of memories is also connected to the current mood, which introduces even more variability to the recollection process [6]. To rectify the disadvantages of the retrospective method, in recent years, researchers and clinicians have begun to adopt different approaches. The methodologies range from the use of real-time electronic diaries, maintained by the user, to the use of sophisticated technology to extract psycho-physiological information independent of the user’s input [4]. However, the efficiency and effectiveness related to application of these ambulatory assessment methods in general psychiatric practice is yet to be observed. This manuscript presents a technology-based system aimed at facilitating self-care for people with bipolar disorder (BD).

The nature of the BD condition is that patients can experience the extremes of low mood and inactivity (depression phase), which then can swing to hyperactivity and grandiosity in the manic phase. Although, BD is not the most widely spread psychiatric condition, reports show that, among mental illnesses, BD alongside of schizophrenia has the highest “disability rating”. This rating a result of apprising particular conditions in terms of severity and length of disability caused regardless of whether it is social, physical of other kind of disability [2]. Therefore, developing novel means of managing the condition is an important, interesting, yet challenging goal for modern health services which potentially can be tackled using means of personalized, pervasive computing.

Research on BD therapies as well as clinical evidence indicates that bipolar patients are a group which is likely to benefit from a personal monitoring and treatment tool. The main reasons are:

· In general, it was proven that even diary based “early warning system” against BD can improve effectiveness of interventions [7]

· The occurrence of opposite extremes of behaviour, characterising the condition, is likely to be apparent in physical and social activities possible to monitor via pervasive technology [8].

· Unlike numerous psychiatric conditions patients with bipolar disorder possess in general a high self-awareness and are willing to comply with treatment regime [8].

· There is a known link between the condition and increased creativity, which renders the patient group as more likely to accept novel approaches to the management regime [9,10].

## Background

Modern developments in sensor technology enable the deployment of ambulatory assessment systems collecting diverse quantitative data, as well as explicit user inputs. Currently there is a wide range of sensing techniques used especially in wellness and health monitoring applications. One group of sensing techniques relates to measuring physical activity and posture. This involves the use of accelerometers, step-counters, gyroscopes etc. Such means are utilised in studies dealing with specific tasks like appraising the progress of Parkinson’s disease [11], Multiple Sclerosis [12], detecting falls [13] or even cough monitoring [14]. Moreover, actigraphy is also used for more general purposes, for example the estimation of energy expenditure and overall activity of the user [15]. These techniques find applications in researching the causes of obesity, stress and other syndromes that could be related to general physical activity [15].

Depending on the application, ambulatory monitoring systems also incorporate numerous sensors acquiring physiological data, and almost every behavioural disorder has its base in a patient’s physiological mechanisms and biological events are important facets of many cognitive problems [16]. Changes in heart rate, blood pressure, cortisol levels and EEG profiles and other vital signs, are often a definite indicator of numerous mental conditions [17]. Therefore, collecting momentary physiological data may appear to be a valid application of ambulatory technology. However, the main barrier to the wide application of physiological measurements in a real-life ambulatory setting is the cumbersomeness of collecting such data on a day-to-day basis. This renders ambulatory physiological sensors impracticable. A more suitable approach is to monitor patients’ activity via other means. The importance of collecting sensor-based data in addition to traditional self-assessment reports is of particular importance in dealing with psychiatric and psychological conditions. Several studies showed that the objective, quantitative data collected via the use of activity or physiological measurements can differ greatly from the subjective perception of a self-reporting patient [18]. In general, researchers and practitioners agree that while questionnaires are undoubtedly suitable as a method for studying subjective attitudes and representations of an experience, they cannot substitute for actual objective behavioural data coming from everyday observance [18]. However, applying sensor-based ambulatory monitoring into psychiatry is a relatively novel field with very few solutions tested and implemented on a wide scale in clinical practise.

These premises constitute the base for the research presented in this manuscript as well as other investigations into utilizing technology in tackling bipolar disorder. Examples of those include the EU Monarca project [19], which origins from earlier works on applying monitoring in facilitating the care for BD patients [20], as well as the PSYCHE project which aims to use smart garments to collect physiological data relevant to mental wellbeing [21]. Both of these implementations aim to identify physiological markers for early signs of an episode. To achieve this, inputs from heart rate to EEG waves would be collected. As such approach may pose an incompliance risk due to discomfort caused by physiological sensors, the methods presented in this manuscript aim to rather collect behavioural data from non-physiological sensors and data collecting techniques.

## Methods

Bipolar disorder is characterised by occurrences of depressive and manic episodes, each with its own specific outcomes. Often the episodes can directly follow each other. The occurrence of an episode results in major behavioural changes, which are apparent in all areas of patient’s life [22]. The syndromes include psychomotor agitation, loud speech, self-deprivation of sleep in manic episodes and psychomotor retardation, indecisiveness, insomnia in the case of depressive ones.

The established practice of assessing the patient’s wellbeing in bipolar disorder is to interview the patient regularly. The cooperation with the clinician and well-developed self-awareness of the person affected by the condition is key to maintaining the desired mood stability. It is also generally agreed that keeping to a constant life routine is a major factor in dealing with the disorder. The areas of life likely to be affected by an onset of an episode can be monitored by pervasive technology. The main research question for a personalized ambient monitoring system is whether electronically aided observation could assist in recognising early signs or detect known triggers of an episode and prompt an intervention aimed at pre-empting an episode and its consequences.

The aim of a sensor network in personalised ambient monitoring is to provide empirical data to be processed in the core of the system. The sensors should be able to observe the key areas affected by the condition and its symptoms. This objective data could then be used to enhance the assessment scale input. The first step was to match expected bipolar symptoms with possible sensing techniques. Such an approach was also adopted by Wihelm and Roth in their study on panic disorder [23]. Another premise is to augment the information expected from assessment scales with an appropriate monitoring technology. Such pairings were made and are shown in Table 1. The pairings were created basing on current diagnostic guidelines [24] as well as consulting psychiatrists and patients. The patient may exhibit only a subset of the listed symptoms. However, the diagnostic criterion for BD is the occurrence of at least four of these factors [24].

**Table 1:** Bipolar syndromes matched with sensors (M – manic episode, D- depressive, M/D – applies to both)

**Type**	**Syndrome**	**Possible sensor**
M/D	Altered sleep patterns - insomnia, hypersomnia, self-deprivation of sleep	Possible to monitor with bed sensors as well as light detectors installed in the patient’s home. Effective monitoring of this sleep patterns is of particular importance. Firstly, disturbed sleep can trigger an onset of an episode [25,26]. Secondly it is an important diagnostic indicator that an episode of either kind is occurring [22].
M	Flight of ideas - increased goal oriented activity, euphoria	Monitoring social activity via, e.g., number of visited places (especially in a patient’s free time), number of calls and text messages and their recipients, Monitoring usage of keyboards and household remote controls should also be included, as buttons are likely to be pressed harder and faster.
M/D	Psychomotor agitation (or retardation)	Body (e.g. wrist) worn accelerometer will detect restless behaviour and increased activity. Motion detection can also be of use.
M	Increased (excessive) social activity	Likely to manifest itself in geospatial and temporal patterns (number of visited places). Patients, in their free time, will visit more unusual places and meet new people. These can be monitored via location (e.g., GPS-based) tracking. Identification of crowded places (e.g. clubs) can be achieved through the patient’s mobile device scanning for other devices [27] or quantifying the noise level of the place where the patient is
M	Talkativeness – a pressure to talk louder	Monitored by microphones designed to extract the pitch and volume of speech (and not the content).
D	Concentration problems – indecisiveness	All activities performed on a computer become only related to work duties (e.g. when using of email and web) and they become slow; monitoring keyboard strokes can show decreased speed of typing. Monitoring of household remote controls may indicate lower use.
D	Lack of interest in social and other activities	Monitoring social activity via, e.g., number of visited places will drop as well as the number of Bluetooth encounters [27].
D	Diminished appetite and loss of weight	Regular weight measurements can be automated as well as basic usage of kitchen appliances being monitored.

The techniques proposed in Table 1 require the use of particular sensors. These can be arranged into two sub-groups depending on the envisioned placement: wearable and environmental. The first refers to sensors preferably attached to the patient’s body or carried by them throughout the day. The latter group constitutes of stationary devices monitoring the user’s home environment and their activity there. Table 2 presents the selected sensors in the said manner. These sensors were then implemented to create a prototype of a personalized monitoring system.

**Table 2:** Sensors constituting the personalised ambient monitoring prototype

**Sensor**	**Details**	**Subgroup**
Accelerometer	Body worn tri-axis accelerometer can facilitate monitoring physical activity, posture and (if worn during sleep) sleep patterns.	Wearable
Global Positioning System	GPS can be used to obtain precise outdoor location. The information can be used to monitor changes in activity.	Wearable
Bluetooth scanning	Monitoring Bluetooth environment can provide insight into social encounters as well as augment the localisation process	Wearable
Light detector	The detector should distinguish between natural and artificial sources of light. Turning the light on and off can be a sign of insomnia, restlessness and other behaviours related to the disorder.	Wearable/Environmental
Remote control devices monitor	An Infra-Red detector capable of determining the speed of pressing buttons on a remote control (see Chapter 3).	Environmental
Door switches	Simple on/off devices to monitor usage of household items and (if placed on cupboards in food preparation areas) to provide information regarding eating habits.	Environmental
Motion detectors	Passive Infra-Red (PIR) devices to monitor indoor mobility as well as unusual activity.	Environmental
Bed sensor	This can be a pressure mat under the bed or a capacitive presence sensor embedded in quilts	Environmental

In the first evaluation phase, the system was put to the test in a technical trial involving healthy volunteers in order to establish the usability and identify any fundamental design flaws that may have occurred. The main objective of the trial was to appraise the sensors’ capability of effectively observing the areas of life influenced by the course of BD and detecting slight changes in these areas. Although, in healthy population the observed variations, can be easily attributed to a known cause (e.g. new fitness regime results in higher physical activity), in BD patients can mean a relapse of an episode of either kind. Nevertheless – the changes occur in both cases and testing sensor performance on a group of healthy volunteers was deemed enough for the initial stage.

The second phase was to interview and recruit potential bipolar patients who were willing to test the proposed solutions. The technical trial resulted in five installations. Both trials were granted ethical approval by the UK Research Ethics Committee and NHS Research and Development office.

### Implementation

**Figure 1:**
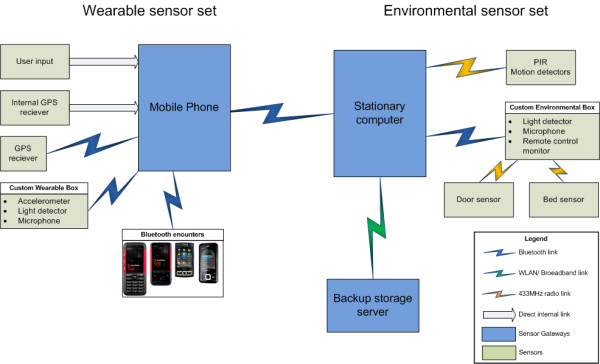
Overview of the sensor network prototype.

As stated above, the implemented sensors can be divided into two groups depending on their target location: environmental i.e. placed in the user’s home and wearable i.e. worn or carried by the user during the day. The first group utilises a stationary computer equipped with commercial and custom-designed software acting as a processing node and storage facility. The latter sensor group is based around a customized mobile phone serving as a processing and storage node. Moreover, in this scheme the phone transfers collected data to the computer for backup. The overview of the implemented prototype is presented in Figure 1.

**Figure 2:**
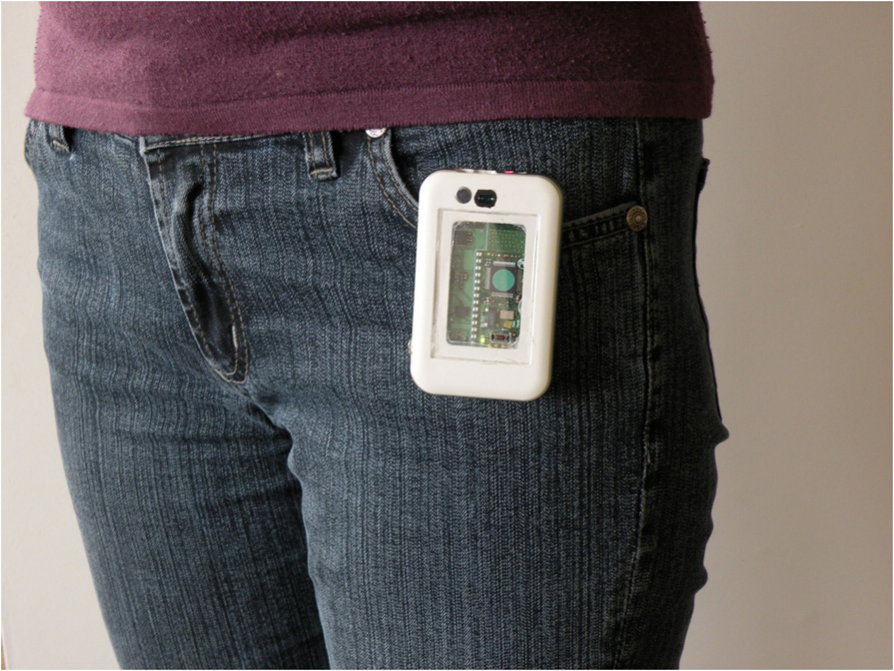
The custom-made belt-worn device incorporating accelerometer, microphone and light sensor.

The wearable sensors consist of an off-the-shelf GPS receiver and a as a belt-worn box (as seen in Figure 2) containing a 3-axis accelerometer with parameters adjusted to observe body movements, light detector capable of distinguishing artificial light and a microphone. Both the GPS receiver and the belt-worn device are Bluetooth enabled to stream data to the hub mobile phone. The phone’s software was modified in order to acquire process and store data from the described devices achieved via a custom-made Java ME applet designed for Java-capable phones. The Bluetooth encounters monitor was a customised applet implemented on the hub phone monitoring for other Bluetooth-enabled devices in the immediate surroundings. Matching their unique identification numbers with particular places and persons (as they might be personal phones of friends and relatives) can give insight into social activity [28]. The GPS position was provided either via the use of a mobile phone’s internal GPS receiver or an external Bluetooth enabled device. Among the advantages of the latter solution was that the overall battery life span was higher compared to that of an internal receiver.

The environmental sensors were contained in a custom-designed box with several simple peripheral sensors. Motion detectors were standard passive infra-red (PIR) devices as used in anti-burglary systems. Door sensors were a simple on/off switches placed in the most used cupboards within the kitchen area whereas the bed usage sensor was a simple pressure mat placed under the bedsheets. The remote control monitor utilised was an infra-red receiver set to detect any standard remote control transmission used in the vast majority of current entertainment equipment. The sensor did not distinguish between particular commands (e.g. changing channel, increasing volume) but treated each as a “remote control event”.

### Participants

A total number of four healthy subjects were monitored at the initial stage of the trial. The participants of this trial were recruited from among people well accustomed with mobile technology. The reasoning for such an arrangement was that the trial objectives were mostly technical and could be addressed more efficiently. All participants were interviewed and gave consent according to the established procedures.

The next stage, following the trial with healthy subjects, was to test out the technology in the end user scenario. This meant inviting BD sufferers to test the technology and share their views on all aspects of the proposed system as well as to participate in a trial similar to the one with healthy volunteers. To facilitate this, a recruitment process was started in the Southampton and Stirling areas. It involved approaching patient self-support groups and charities. Following large initial interest during the process, three participants agreed to the initial home visit but only one participant proceeded with the full installation as others withdrew before the second visit. The individuals did not disclose their reasons for not participating and no further contact was made following their decision in agreement with the ethical guidance obtained.

## Results

The main objective of the trial was to evaluate the performance of the sensor network as well as to assess their potential for monitoring behavioural patterns in BD patients. Therefore the collected data was pre-analysed in order to determine whether the particular sensor carried information that may be fed further into a processing algorithm capable of recognizing the early signs of bipolar episode. Further sub-sections provide a brief overview of the trial results for wearable and environmental sensors as well as providing insight into general participant compliance to monitoring regimes. The presented results come from the trial involving healthy volunteers. The results of the trial involving the BD patient are covered in a separate section below.

### Wearable sensors

**Figure 3:**
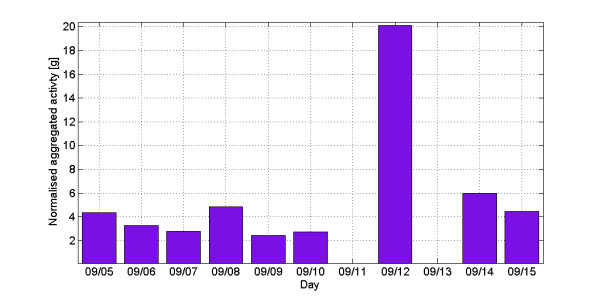
Daily activity based on acceleration.

Each of the wearable sensors included in the set provided valuable behavioural data. The belt-worn accelerometer utilised in the technical trial, as expected, provided information about the subject’s physical activity. It was achieved via subjecting the raw data resulting from long-term monitoring to a three step algorithm integrating overall acceleration over a certain period of time – a methodology widely used in actigraphy [15]. An example of processed data in Figure 3 shows such aggregated activity over the course of two weeks. Missing bars represent days where the sensor was not worn (amount of data was insufficient). Nevertheless, the differences in activity of the user can be observed. In particular the user-reported Sunday workout on 09/12 is apparent.

**Figure 4:**
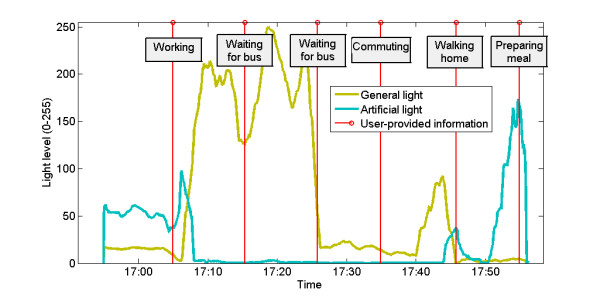
Wearable light sensor and daily activity.

The wearable light sensor utilized in the study was capable of distinguishing between natural and artificial sources of light [29]. Its readings were characterised by periods of high natural light level, which can be accounted for by the user being outdoors in direct sunlight, and periods where dominance of artificial light is apparent, which indicates the user’s presence indoors. This dependence can be observed when data is presented against user self-reports. An example of such is presented in Figure 4.

**Figure 5:**
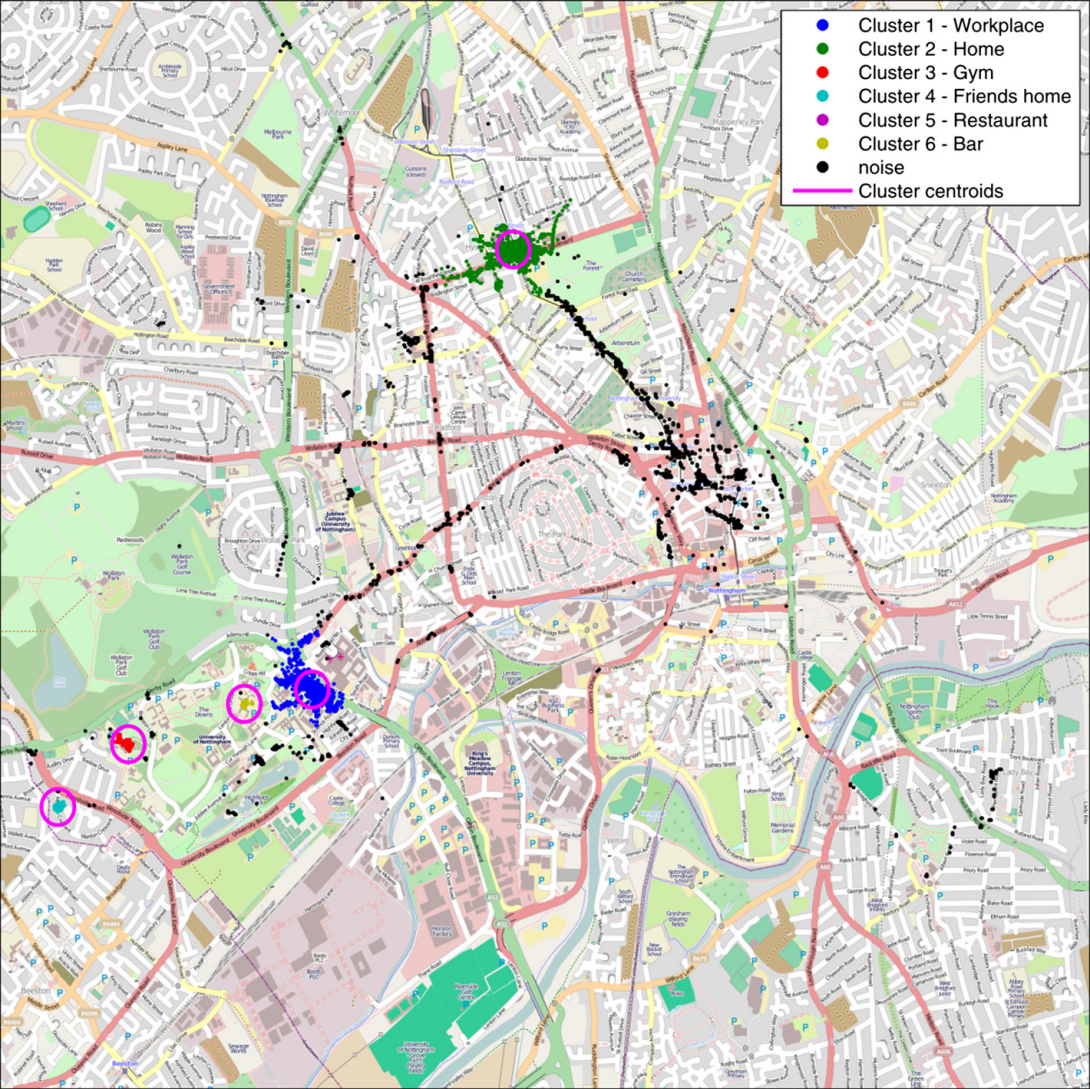
Processed GPS tracks showing identified significant locations.

Location data acquired via the GPS receiver was subjected to clustering aiming to identify user’s meaningful locations. The GPS data was subjected to pre-processing. Firstly, all unreliable points with low satellite coverage were removed. Secondly, since the main aim of the clustering process was to detect significant locations rather than map the journeys between them, another pre-clustering step was to eliminate points where the recorded speed (provided by the GPS receiver using its internal calculations) suggested the subject was moving. The remaining points were subjected to clustering using a density based algorithm (DBSCAN). This facilitated the discovery of meaningful locations where point density was directly related to frequency of visits to such location. Figure 5. presents an example of such clustering. Further details of the GPS data processing methodology can be found in [28] and [27].

The analysis of Bluetooth encounters patterns provided three key information types [27,28]:

· Identifying the most frequently encountered devices, can provide insight into the number and times of interaction with users of these devices occurred.

· Frequently encountered devices can be often tied to a one particular location. Therefore, such encounters can be useful in enhancing the positioning process where GPS localisation is not available (especially indoors).

· The number of encounters in a single scan can indicate a crowded location like bus or a city centre. This kind of information can also be useful in appraising the participant’s social activity.

### Environmental sensors

**Figure 6:**
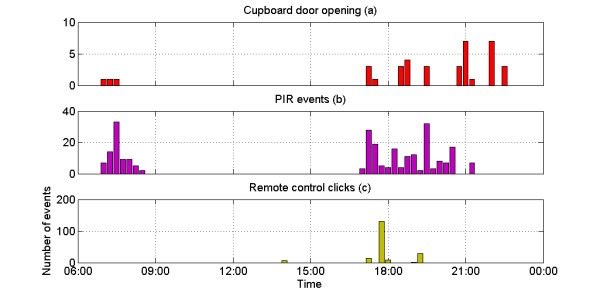
Environmental sensor readings during a typical day.

The environmental sensors included in the trial consisted of PIR motion sensors, door switches and a remote control usage monitor. Each of the devices was activated by an event (e.g. pressing remote control button) with the exact time of these events then stored within the system. Figure 6 shows a set of readings representative of a day of monitoring for one of the participants. Each bar represents an aggregated number of events during a 15 minute interval. In all cases the results corresponded with self-reporting.

**Figure 7:**
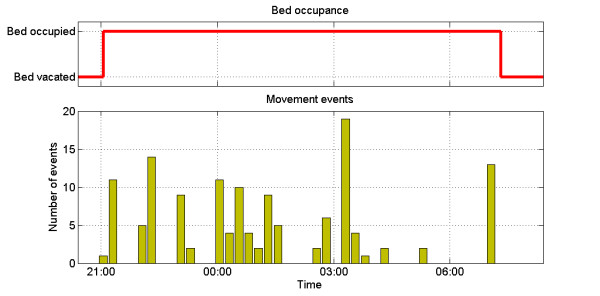
Regular sleep cycle.

**Figure 8:**
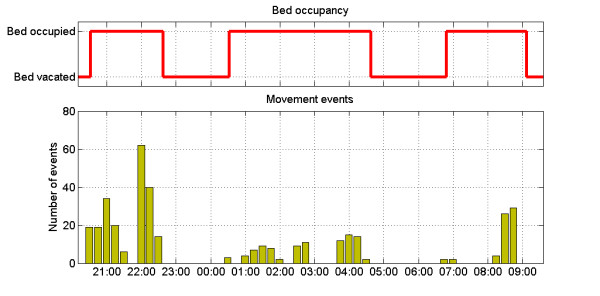
Disturbed sleep cycle with increased number of movement events indicating restlessness.

Additionally, monitoring sleep patterns is of particular importance in managing the course of BD. The sensor utilized in the study, produced two outputs shown in Figures 7 and 8 below. The top part of each figure indicates the user’s presence in the bed. The bottom one shows the number of pressed/un-pressed events which are likely to be caused by the user’s muscle activity during the sleep phases. Each bar represents number of such events during 15 minute long intervals. Increased number of these can indicate restlessness or night terrors – a key indicator of depression [22]. Figure 7 shows a regular sleeping cycle whereas Figure 8 presents data collected during a disturbed sleep for one of the participant.

### Patient trial results

In terms of sensory data, the patient trial produced activity data similar to the sets obtained from the main trial. Since the patient remained euthymic (asymptomatic) throughout the monitoring period, there were no major aberrations from her usual routine. However, the data collected by the wearable node, which depends most on user compliance with a maintenance regime, was far scarcer than from any participant of the technical trial. In one of the intermediate interviews, the patient addressed the issue of not adhering to the routine citing the following reasons:

· Discomfort of carrying extra devices in addition to the mobile phone.

· Forgetfulness regarding charging the sensors, phone or manually starting the monitoring application.

· Lack of familiarity with personal technology in general (not only the PAM system) resulting in underdeveloped user habits.

**Figure 9:**
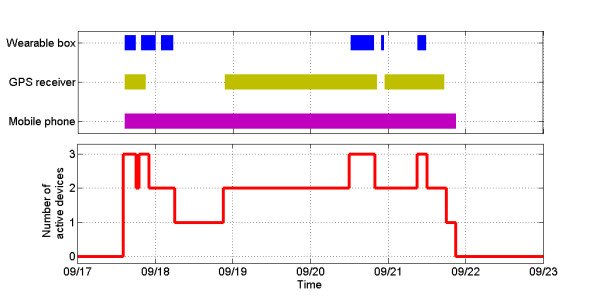
Uplink times for elements of the wearable setup.

Figure 9 presents a summary of how well the participant adhered to maintain separate elements of the wearable setup. The figure shows the uplink times for the devices during a period of most intensive usage of the wearable setup.

It can be observed that while the phone was on and monitoring, the remaining elements were either switched off or remained out of Bluetooth range during extended periods. This was typically due to the user carrying the phone but not the rest of the setup.

### Problems and limitations

As far as the environmental setup did not cause any discomfort to the users, the wearable sensors, although providing large quantities of behavioural data, exhibited significant periods of inactivity. This relates to the issue of user adherence as all participating users reported a level of discomfort caused by the number of devices that needed to be carried. The fact that the preferred placement of the wearable node should provide it with exposure to the ambient light sources, only added to the problem.

Overall, the main limitation of the work presented in this manuscript is its relatively small scale of the trials. The cohort of participants is too small to derive a detailed behaviour monitoring scheme. Moreover, the high rejection rate among the interviewed patients indicates that the system may present itself as too obtrusive. However, the general notions regarding compliance issues and observations regarding the collected sensory data remain valid and constitute a good base for further investigations in the field.

## Conclusions

In this paper we describe the deployment and evaluation of a personalised ambient monitoring prototype that included several sensory devices either worn by the users or placed in their home environment for a purpose of monitoring of onsets of bipolar disorders. Every evaluated element provided potentially useful data about the users’ activity and behavioural patterns. However, the main and most persistent issue discovered throughout the tests was the impracticality of maintaining and “wearing” as many devices on a day to day basis. Such concerns did not apply to environmental sensors as they require minimal maintenance and, aside from the bed usage sensor, caused neither discomfort nor unease. Considering this, a successful wearable monitoring system should not interfere with the user’s routine and habits. Therefore pursuing wearable monitoring via only a mobile phone in connection with a set of simple unobtrusive environmental sensors is a logical choice for further investigations.

Nevertheless, the main conclusion arising from this and similar studies in the field is that despite the technology being developed and sophisticated enough to provide independent insight into patient’s behaviour, psychiatrists and psychologists are yet to adopt such means into their general practise. This contrasts with other fields of medicine where the use of monitoring and diagnostic technology is well established.

Overcoming this barrier requires cooperation between researchers, clinicians and policymakers. Research needs to involve and inform the psychiatric community of positive outcomes of implemented studies. Only then can modern technology-based approaches to mental care can be successfully rolled out and their true value appraised.

## Competing interests

The authors declare that they have no competing interests.

## Authors’ contribution

PP and JC devised the study and its elements, whereas KW provided background information and information on user needs. All authors read and approved the final manuscript.
